# Using molecular transmission networks to understand the epidemic characteristics of HIV-1 CRF08_BC across China

**DOI:** 10.1080/22221751.2021.1899056

**Published:** 2021-03-22

**Authors:** Kang Li, Meiliang Liu, Huanhuan Chen, Jianjun Li, Yanling Liang, Yi Feng, Hui Xing, Yiming Shao

**Affiliations:** a Key Laboratory of Molecular Microbiology and Technology, Ministry of Education, College of Life Sciences, Nankai University, Tianjin, People’s Republic of China; b State Key Laboratory for Infectious Disease Prevention and Control, National Center for AIDS/STD Control and Prevention, Chinese Center for Disease Control and Prevention, China CDC, Beijing, People’s Republic of China; c Guangxi Key Laboratory of AIDS Prevention and Treatment & Guangxi Universities Key Laboratory of Prevention and Control of Highly Prevalent Disease, School of Public Health, Guangxi Medical University, Nanning, People’s Republic of China; d Guangxi Center for Disease Prevention and Control, Nanning, People’s Republic of China

**Keywords:** HIV-1, CRF08_BC, molecular epidemiology, risk factors, transmission cluster

## Abstract

HIV-1 CRF08_BC has become a major epidemic in heterosexuals and intravenous drug users (IDUs) in southern China. In order to evaluate the trends of its epidemic and facilitate targeted HIV prevention, we constructed the genetic transmission networks based on its *pol* sequences, derived from the National HIV Molecular Epidemiology Survey. Through retrospective network analysis, to study the epidemiological and demographic correlations with the transmission network. Of the 1,829 study subjects, 639 (34.9%) were clustered in 151 transmission networks. Factors associated with increased clustering include IDUs, heterosexual men, young adults and people with lower education (*P* < 0.05 for all). The IDUs, MSM, young adult and person with low education had more potential transmission links as well (*P* < 0.05 for all). The most crossover links were found between heterosexual women and IDUs, with 30.9% heterosexual women linked to IDUs. The crossover links heterosexual women were mainly those with middle age and single (*P* < 0.001). This study indicated that the HIV-1 CRF08_BC epidemic was still on going in China with more than one third of the infected people clustered in the transmission networks. Meanwhile, the study could help identify the active CRF08_BC spreader in the local community and greatly facilitate précising AIDS prevention with targeted intervention.

## Introduction

Inferring human immunodeficiency virus (HIV) molecular networks via sequence analysis can provide insight into the dynamics of viral transmission in populations and sub-populations [1–4]. As genetic similarity between HIV sequences generated from two persons with HIV, it suggests that these people likely belong to the same transmission network [5–7]. Previous studies using these molecular transmission methods have: revealed transmission routes, predisposing factors and epidemiologic linkages [6], and inform prevention efforts [2,8,9].

As the HIV epidemic in China has diversified, many new subtypes have been introduced and new circulating recombinant forms have been created. Currently, the four major strains, CFR01_AE, CRF07_BC, CRF08_BC and B’ (Thailand variant of subtype B), cause more than 90% of all infections in China [1,10]. It is noteworthy that CRF08_BC has been involved in many newly reported new HIV-1 unique recombinant form by further recombination with other strains [11–14]. Previous local molecular epidemiology studies have also found that CRF08_BC has become one of the predominant subtypes among people with injection drug use and heterosexual risk, especially in the southwestern and eastern China [11,13,15]. These CRF08_BC studies have been largely cross-sectional [16,17]. Thus, pattern of transmission of CRF08_BC across all of China remain largely unknown and can affect the feasibility of interventions.

This study used China nationwide epidemiology survey and HIV sequence data to: infer a longitudinal HIV-1 CRF08_BC transmission network for the first time, provide an insight with depth in CRF08_BC transmission between risk populations, and elucidate potential links among risk networks. The results of these analyses can be used to provide effective information for HIV prevention efforts.

## Materials and methods

### Sources of sequence data

All available HIV-1 CRF08_BC sequences covering the entire protease (PR) and partial reverse transcriptase (RT) region (HXB2 genome location 2253–3554) were collected from the China National Center for AIDS/STD Control and Prevention (NCAIDS) molecular database (the previous investigations was conducted in a cross-sectional method using stratified random sampling by province), moreover, partial sequences were downloaded from Los Alamos HIV sequence database (LANL, http://www.hiv.lanl.gov, default parameters, last updated: January 2018). Our analysis included one sequence per person in the database. When more than one sequence was available (4.1% of persons) only the HIV sequence obtained from the earliest time point was included. We performed a BLAST search by using the collected sequence and selected top 10 sequences with the highest homology with the references for each sequence which was further determined the subtype through phylogenetic tree analysis and excluding repeated sequence [18,19]. Since network inference is less precise with shorter sequences, we eliminated sequences that were less than 900 nucleotides in length [5,20].

### Inference of molecular network

To infer the molecular network, the genetic distance, Tamura-Nei 93 nucleotide substitution model (TN93) was used to calculate pairwise distance between all pairs of HIV *pol* sequences using HYPHY version 2.2.4 [1,10,21]. Using TN93 distance to compute single nucleotide transversion rates and 2 nucleotide conversion rates, as they are the sophisticated genetic distances, they can be represented by closed-form solutions that allow fast distance calculations [22]. We conducted an initial analysis using a genetic distance threshold of 0.7% substitutions/site, since this distance discriminated the maximum number of clusters and has a relatively high resolution in the genetic network (Supplementary Figure S1) [7, 20]. And those conform with the criterion were deemed to have potential transmission relation. The network data were visualized using a custom R script in the network package in the R software version 3.5.1.

### Analysis of molecular network characteristics

After the network was inferred, we analysed characteristics of the transmission network, including the number of sequences (nodes), links (edges), and the degrees which each individual in the network was defined as the number of links with other individuals. Correlates of clustering were investigated using bivariate analyses and multivariable logistic regression models, and the groups analysed included both all individuals and clustering versus not clustering individuals [10,23]. The topological properties and parameters of the CRF08_BC networks (e.g. nodes, edges, density, and clustering coefficients) were computed with the Cytoscape version 3.2.0 NetworkAnalyzer tool (https://med.bioinf.mpi-inf.mpg.de/netanalyzer/index.php) [24,25].

Furthermore, we observed the annual dynamics of the significant dense clusters (stationary/expansion) during 2000–2017. For this purpose, modular framework and clusters of tightly interconnected nodes were identified using the Molecular Complex Detection (MCODE) 1.3 plug-in application with default parameters (including degree cut-off of 2, node score cut-off of 0.2, K-core of 2, and maximum depth from seed of 100). This rank and score larger more dense complexes higher in the molecular network results [26–28]. Meanwhile, in order to elucidate the effect of the clusters of different growth patterns of persons living with HIV to the epidemic situation, we can define the high-transmission speed clusters and low-transmission speed clusters according to the size and increment situation of the sub-cluster [29].

### Analysis of individuals with multiple potential transmission links

In the network, two groups of people were compared including: individuals who were linked to 1–3 other persons and individuals who linked to ≥4 others (i.e. those in the highest quartile of those persons with any links) [6,30,31]. Multivariable logistic regression was applied to determine factors associated with having ≥4 links. The model included age at HIV diagnosis, sampling year, education, marital status, transmission category, and domicile of report.

### Analysis of mixing between people reporting different transmission risk

To understand the characteristics of links between persons of the different risk groups and analyse the potential transmission partners, we explored (1) the number of individuals who have links to different risk groups in the transmission networks and (2) the characteristics of crossover persons. All analyses were stratified by transmission categories (including heterosexual contact, persons who inject drugs, male-to-male sexual contact and unknown), geographical distribution (including eastern, northern, central, and southwestern China), age (stratified into <20, 20–29, 30–39, 40–49, 50–59, and ≥60), education level (including primary school, middle school, high school, and above college), marital status (including single, married, and divorced or widowed), and the information not reported or not identified was classified as unknown. The chi-square tests (*χ*^2^) and Fisher exact tests were applied to examine differences between subgroups. These datasets were analysed in SPSS version 22.

## Results

### Study population

A total of 1829 HIV-1 CRF08_BC *pol* sequences (including NCAIDS database of 1504, LANL database, 325) were available between 2000 and 2017 and were evaluated from the following regions: 293 Eastern, 32 Northern, 122 Central, and 1382 Southwestern in China. Self-reported HIV risk factors included: 1233 (67.4%) heterosexual, 69 (3.8%) men who have sex with men (MSM), 426 (23.3%) IDUs, and 8 (0.4%) blood transfusion (BT). Furthermore, of the persons with sequences included in this analysis, 1087 (59.4%) were male, 1332 (72.8%) were Han ethnicity, 685 (37.5%) were middle school education level, and 497 (27.2) % were aged 20–39 years (Table 1).

**Table 1.** Demographic and clustering characteristics of the study subjects.

		Total (column %)	Clustering (row %)	Not clustering (row %)
All		1829 (100.0)	639 (34.9)	1190 (65.1)
Transmission risk (birth sex)	Hetero (F)	524 (28.6)	149 (28.4)	375 (71.6)
	Hetero (M)	705 (38.5)	243 (34.5)	462 (65.5)
	Hetero (U)	4 (0.2)	2 (50.0)	2 (50.0)
	MSM	69 (3.8)	13 (18.8)	56 (81.2)
	IDUs	426 (23.3)	207 (48.6)	219 (51.4)
	BT	8 (0.4)	2 (25.0)	6 (75.0)
	Unknown	93 (5.1)	23 (24.7)	70 (75.3)
Region	Central	122 (6.7)	26 (21.3)	96 (78.7)
	North	32 (1.7)	11 (34.4)	21 (65.6)
	East	293 (16.0)	102 (34.8)	191 (65.2)
	Southwest	1382 (75.6)	500 (36.2)	882 (63.8)
Sampling year	2000–2007	304 (16.6)	193 (63.5)	111 (36.5)
	2008–2011	483 (26.4)	173 (35.8)	310 (64.2)
	2012–2015	695 (38.0)	147 (21.2)	548 (78.8)
	2016–2017	347 (19.0)	126 (36.3)	221 (63.7)
Education level	Primary school	471 (25.8)	174 (36.9)	297 (63.1)
	Middle school	685 (37.5)	234 (34.2)	451 (65.8)
	High school	302 (16.5)	111 (36.8)	191 (63.2)
	College or higher	190 (10.4)	55 (28.9)	135 (71.1)
	Unknown	181 (9.9)	65 (35.9)	116 (64.1)
Age at diagnosis	<20	61 (3.3)	16 (26.2)	45 (73.8)
	20–29	505 (27.6)	148 (29.3)	357 (70.7)
	30–39	459 (25.1)	190 (41.4)	269 (58.6)
	40–49	316 (17.3)	129 (40.8)	187 (59.2)
	50–59	164 (9.0)	51 (31.1)	113 (68.9)
	≥60	183 (10.0)	62 (33.9)	121 (66.1)
	Unknown	141 (7.7)	43 (30.5)	98 (69.5)
Marital status	Single	793 (43.4)	275 (34.7)	518 (65.3)
	Married	739 (40.4)	265 (35.9)	474 (64.1)
	Divorced or widowed	120 (6.6)	34 (28.3)	86 (71.7)
	Unknown	177 (9.7)	65 (36.7)	112 (63.3)

Abbreviations: Hetero, heterosexual; MSM, men who have sex with men; IDUs, intravenous drug users; BT, blood transfusion; Unknown, data are not available.

### Characteristics of transmission networks

**Figure 1.**
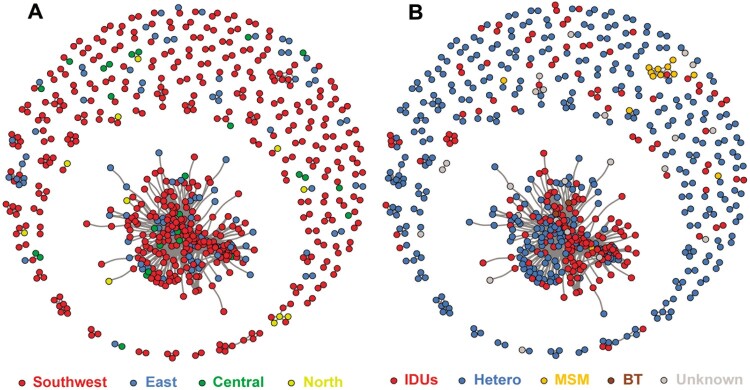
China HIV-1 CRF08-BC surveillance genetic transmission networks. Nodes indicate one sequence or individual. Edge (i.e. links) represent genetic linkage (≤0.007 substitutions/site). Colour indicates different transmission risk factor and different regions of China. Abbreviations: Hetero, heterosexual; MSM, men who have sex with men; IDUs, intravenous drug users; BT, blood transfusion; Unknown, data are not available.

Under the threshold of 0.7% genetic distance, 639 nodes linked to at least one other sequence, thus considered as clustered in the inferred molecular network (Supplementary Figure S1). This resulted in 152 clusters ranging in size from 2 to 254 sequences with a total of 2144 links. The number of links per sequence ranged from 1 to 133 (median: 1, interquartile range: 1–4). Figure 1 illustrates the region and risk-specific distribution of HIV-1 CRF08_BC. In all clusters within the molecular network, people reporting IDUs and heterosexual risk were the largest risk groups, accounting for 207 (32.4%) and 394 (61.7%) of nodes, respectively. People with MSM risk accounted for only 13 (2.0%) nodes, and people with BT and Unknown risk together accounted for only, 25 (3.9%) of the clustering nodes. CRF08_BC infections were the most concentrated in the southwestern region (500, 78.2%), followed by eastern (102, 16.0%), central (26, 4.1%), and northern (11, 1.7%). Furthermore, there was one large cluster in the network (*n *=* *254 persons) which mainly belonged to southwestern and eastern region sequences, and the majority of the persons in this cluster reported IDUs and heterosexual risks (Table 1).

### Characterizing network growth

**Figure 2.**
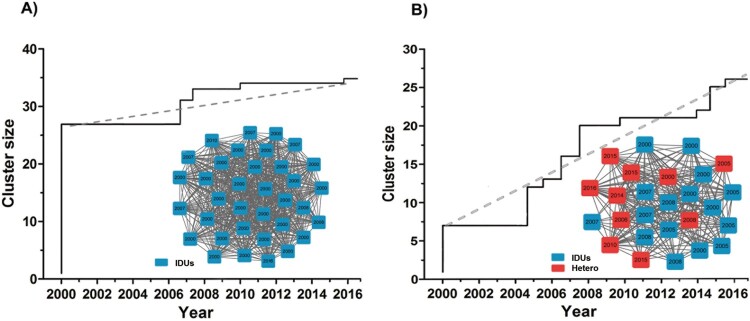
Different cluster growth dynamics of CRF08_BC network. This image displays the two-growth pattern of the network. The *X*-axis time interval is year, the *Y*-axis is the value of the cluster size. Colour indicates different transmission risk factor. Abbreviations: Hetero, heterosexual; IDUs, intravenous drug users. The dotted grey line represents the straight line fitted by each point.

To clarify the dynamic changes of the main CRF08_BC subtype molecular network, MCODE analysis identified two significant modules clusters with network scores ranging between 33.6 and 19.4. Therefore, we investigated the cluster changes in a subgroup analysis. As shown in Figure 2, the type A cluster which had 35 nodes and 572 edges, however, only has added 8 new nodes from 2000 to 2017. The proportion of new clusters in the transmission cluster was significantly reduced after 2010 and it belongs to the low-growth network. Of note, almost all of the new individuals belong to IDUs risk category. These networks have gradually weakened the impact on the epidemic situation. Additionally, the IDUs and heterosexual groups constituted the type B cluster with 26 nodes and 242 edges. The cluster grew from 7 nodes in 2000 to 26 nodes in 2017. Number of cluster new cases increased year by year and it belongs to the high-growth network, and its influence on the epidemic has progressively increased.

### Factors correlated with clustering

Next, we explored which demographics and reported risks factors associated with clustering. In bivariate analyses (Supplementary Table S1), individuals whose sequences clustered were more likely to be in the heterosexual or IDU risk factor group, the middle-aged person and the persons with lower education. As expect, in multivariable analysis (Table 2), increased odds of clustering were indicated among persons reporting IDUs risk, men reporting heterosexual risk, people diagnosed between 30 and 49 years of age, and the persons with a lower level of education. Further, the clustering frequency differed across China with persons who resided in southwestern and eastern China at diagnosis had significantly higher odds of clustering. Moreover, participants from northern and central regions were less likely to fall into clusters than participants from the other sites, probably due to the fewer genotypes of HIV-1 CRF08_BC (lower subtype density) in these locations, rather than differences in transmission risk. The nodes sampled after 2008 and the MSM risk factor were significantly less likely to cluster (all *P *<* *.05). There was no difference in proportion clustering by ethnicity and marital status.

**Table 2.** Factors associated with clustering in the CRF08_BC transmission network in multivariate logistic regression model.

Attribute	Category	Likelihood of clustering, adjusted odds ratio	Odds ratio 95% CI	*P*-value
Transmission risk (birth sex)	Hetero (F)	–	–	–
	Hetero (M)	1.32	1.03–1.68	.029
	Hetero (U)	2.49	0.35–17.82	.365
	MSM	0.50	0.25–0.98	.043
	IDUs	2.34	1.79–3.07	<.001
	BT	0.85	0.17–4.28	.847
	Unknown	0.81	0.49–1.35	.414
Region	Central	–	–	–
	North	1.94	0.82–4.56	.129
	East	1.89	1.15–3.12	.012
	Southwest	2.04	1.31–3.2	.002
Sampling year	2000–2007	–	–	–
	2008–2011	0.33	0.24–0.44	<.001
	2012–2015	0.16	0.12–0.21	<.001
	2016–2017	0.34	0.24–0.47	<.001
Education level	Primary school	–	–	–
	Middle school	0.69	0.54–0.88	.003
	High school	0.86	0.64–1.15	.301
	College or higher	0.59	0.41–0.85	.004
	Unknown	0.81	0.57–1.15	.238
Age at diagnosis	<20	–	–	–
	20–29	1.18	0.65–2.16	.586
	30–39	1.96	1.08–3.58	.028
	40–49	1.93	1.05–3.57	.035
	50–59	1.29	0.66–2.49	.455
	≥60	1.48	0.78–2.84	.233
	Unknown	1.23	0.63–2.42	.541
Marital status	Single	–	–	–
	Married	1.04	0.84–1.28	.739
	Divorced or widowed	0.70	0.45–1.10	.121
	Unknown	0.87	0.57–1.33	.529

Abbreviations: Hetero, heterosexual; MSM, men who have sex with men; IDUs, intravenous drug users; BT, blood transfusion; Unknown, data are not available; CI, confidence interval.

### Nodes with multiple potential transmission links

**Figure 3.**
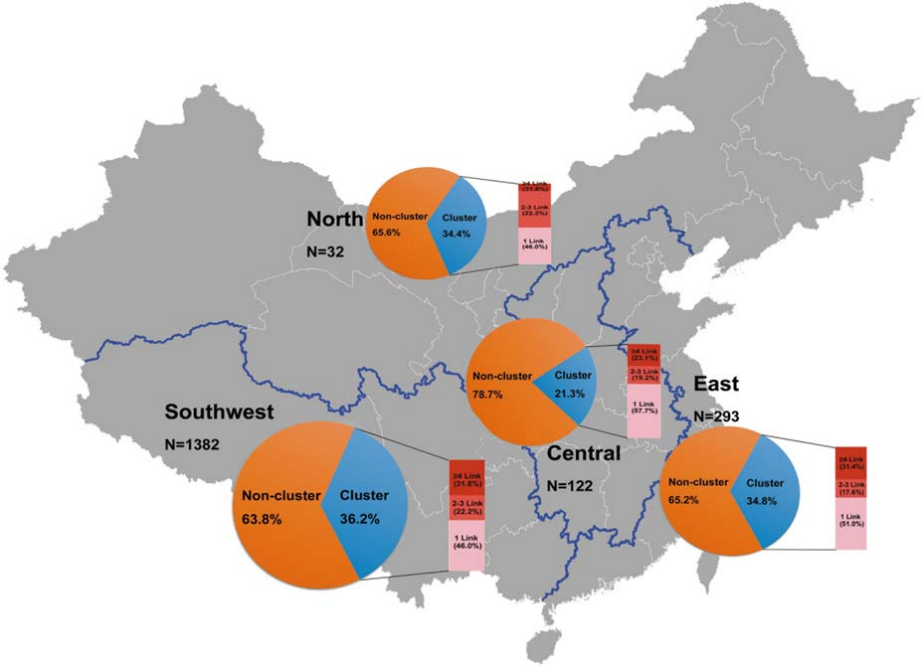
Map of HIV-1 CRF08_BC population distribution in different regions in mainland China, percentage of clustering by region, and proportion of nodes with multiple potential transmission links.

On the whole, 2144 links were constructed by 639 nodes in this transmission networks, among which 47.3% included 1 link, 21.8% included 2–3 links, and 31.0% had ≥4 links (Figure 3). Individuals with ≥4 links were more likely to be aged 30–39 years (*P *=* *.003), the persons with lower education (*P *<* *.001) and report IDUs/MSM risk factor (*P *<* *.05, *P *=* *.046; Table 3). Similar results were seen using a bivariate logistic regression model, except that the education level of senior high school and the MSM risk factor were marginally associated with ≥4 links clustering (*P *=* *.14 and .06; Supplementary Table S2). Overall, although these individuals with ≥4 links represented only 198 of 639 individuals (31.0%), they were extensively involved in the vast majority links (85.0%) of the CRF08_BC network.

**Table 3.** Factors associated with potential transmission links.

Attribute	Category	Total	1–3 Links, *n* (%)	≥4 Links, *n* (%)	Adjusted odds ratio	Odds ratio 95% CI	*P*-value
All		639	441(69.0)	198(31.0)	–	–	–
Transmission risk (birth sex)	Hetero (F)	149	119(79.9)	30(20.1)	–	–	–
	Hetero (M)	243	196(80.7)	47(19.3)	1.14	0.5–2.62	.758
	Hetero (U)	2	1(50.0)	1(50.0)	1.32	0.04–42.47	.877
	MSM	13	7(53.8)	6(46.2)	5.22	1.03–26.56	.046
	IDUs	207	97(46.9)	110(53.1)	5.17	2.13–12.52	<.001
	BT	2	2 (100.0)	0 (0.0)	0.00	N/A	.999
	Unknown	23	19 (82.6)	4 (17.4)	0.68	0.21–2.24	.526
Region	Central	26	20 (76.9)	6 (23.1)	–	–	–
	North	11	10 (90.9)	1 (9.1)	0.18	0.02–1.81	.145
	East	102	70 (68.6)	32(31.4)	0.90	0.32–2.55	.846
	Southwest	500	341 (68.2)	159 (31.8)	1.09	0.42–2.85	.853
Sampling Year	2000–2007	193	100 (51.8)	93 (48.2)	–	–	–
	2008–2011	173	113 (65.3)	60 (34.7)	0.71	0.4–1.25	.236
	2012–2015	147	127 (86.4)	20 (13.6)	0.26	0.13–0.55	<.001
	2016–2017	126	101 (80.2)	25 (19.8)	0.31	0.15–0.64	<.001
Education level	Primary school	174	113 (64.9)	61 (35.1)	–	–	–
	Middle school	234	164 (70.1)	70 (29.9)	0.39	0.22–0.67	<.001
	High school	111	74 (66.7)	37 (33.3)	0.52	0.28–0.96	.038
	College or higher	55	38 (69.1)	17 (30.9)	0.44	0.19–1.01	.053
	Unknown	65	52 (80.0)	13 (20.0)	0.25	0.08–0.78	.017
Age at diagnosis	<20	16	11 (68.8)	5 (31.3)	–	–	–
	20–29	148	103 (69.6)	45 (30.4)	0.75	0.23–2.47	.633
	30–39	190	116 (61.1)	74 (38.9)	5.78	1.8–18.54	.003
	40–49	129	82 (63.6)	47 (36.4)	1.38	0.26–2.98	.847
	50–59	51	42 (82.4)	9 (17.6)	0.31	0.07–1.4	.130
	≥60	62	48 (77.4)	14 (22.6)	1.42	0.36–5.58	.619
	Unknown	43	39 (90.7)	4 (9.3)	0.00	N/A	.999
Marital status	Single	275	187 (68.0)	88 (32.0)	–	–	–
	Married	265	180 (67.9)	85 (32.1)	0.89	0.55–1.45	.651
	Divorced or widowed	34	24 (70.6)	10 (29.4)	1.44	0.52–4.01	.484
	Unknown	65	50 (76.9)	15 (23.1)	0.62	0.23–1.7	.358

Abbreviations: Hetero, heterosexual; MSM, men who have sex with men; IDUs, intravenous drug users; BT, blood transfusion; Unknown, data are not available; CI, confidence interval.

### Transmission links mixing by risk category

Among persons reporting a history of injection drug use, 81.2% were more commonly linked to other persons reporting IDUs risk, meanwhile, some were also linked to women reporting heterosexual risk (45.4%; Table 4). For the heterosexual risk groups of CRF08_BC: men reporting heterosexual risk were most commonly linked to women reporting heterosexual risk (48.9%) and few were found to be linked with MSM (2.1%) and IDUs (17.3%); a majority of women reporting heterosexual generally risk shared links with men reporting heterosexual risk (75.8%), however, many were also linked to other IDUs (30.9%). We can see that the various risk categories have individuals linked to drug use. Then, we investigated links between IDUs and heterosexual women groups (Table 5). There were significant age differences, the percentage linked to IDUs was highest for aged 30–39 years (48.8%) and 40–49 years (33.3%), *P *=* *.037. Additionally, concerning the marital status factors, a higher percentage of persons reporting being unmarried (37.3%) were linked to persons reporting IDUs risk, as compared to married (23.0%), *P *=* *.047. Further, nearly half of people reporting IDUs (45.4%) risk were linked to the individuals reporting heterosexual women risk. However, this percentage changed remarkably by age, IDUs aged 30–39 years had the highest percentage (78.5%) linked to heterosexual women than among those in the other age groups, *P *<* *.001. It did not significantly fluctuate by region, education level and marital status factors.

**Table 4.** Number of individuals who have links to different risk groups in the transmission networks.

Risk groups	All	Hetero men	Hetero women	UN hetero	IDUs	MSM	BT	Unknown	*P*-value^a^
Hetero men	243	162 (66.7)	119 (48.9)	1 (0.4)	42 (17.3)	5 (2.1)	2 (0.8)	9 (3.7)	<.001
Hetero women	149	113 (75.8)	102 (68.5)	3 (2.0)	46 (30.9)	1 (0.7)	1 (0.7)	11 (7.4)	<.001
IDUs	207	71 (34.4)	94 (45.4)	1 (0.5)	168 (81.2)	1 (0.5)	0 (0.0)	57 (27.5)	<.001
MSM	13	6 (46.2)	1 (7.7)	0 (0.0)	1 (7.7)	9 (69.2)	0 (0.0)	0 (0.0)	<.001

Note: One person can have links to more than one risk group. Abbreviations: Hetero, heterosexual; MSM, men who have sex with men; IDUs, intravenous drug users; BT, blood transfusion; Unknown, data are not available; CI, confidence interval.

^a^ Chi-square trend test.

**Table 5.** Characteristics of links between heterosexual women and IDUs in the network.

Attribute	Category	Number of hetero women	Linking to IDUs, *n* (%)	chi-square *P*-value	Number of IDUs	Linking to hetero women, *n* (%)	chi-square *P*-value
Total		149	46 (30.9)		207	94 (45.4)	
Education level	Primary school	46	17 (37.0)	.777	63	25 (39.7)	.460
	Middle school	66	19 (28.8)		58	28 (48.3)	
	High school	23	7 (30.4)		46	23 (50.0)	
	College or higher	11	2 (18.2)		9	6 (66.7)	
	Unknown	3	1 (33.3)		31	12 (38.7)	
Region	Central	12	5 (41.7)	.510	3	1 (33.3)	.972
	North	2	1 (50.0)		2	1 (50.0)	
	East	17	7 (41.2)		43	19 (44.2)	
	Southwest	118	33 (28.0)		159	73 (45.9)	
Age at diagnosis	<20	11	2 (18.2)	.037	4	3 (75.0)	<.001
	20–29	53	10 (18.9)		44	8 (18.2)	
	30–39	41	20 (48.8)		65	51 (78.5)	
	40–49	18	6 (33.3)		49	21 (42.9)	
	50–59	19	6 (31.6)		17	2 (11.8)	
	≥60	6	1 (16.7)		10	2 (20.0)	
	Unknown	1	1 (100.0)		18	7 (38.9)	
Marital status	Single	67	25 (37.3)	.047	95	43 (45.3)	.928
	Married	74	17 (23.0)		72	32 (44.4)	
	Divorced or widowed	6	2 (33.3)		5	3 (60.0)	
	Unknown	2	2 (100.0)		35	16 (45.7)	

Abbreviations: Hetero, heterosexual; IDUs, intravenous drug users; Unknown, data are not available.

## Discussion

Since the HIV-1 CRF08_BC initial introduction to China in 1997 [32], it had a nontrivial impact on China public health. Previous studies ordinarily have focused on the HIV-1 CRF01_AE and CRF07_BC epidemics in China, but not CRF08_BC [33–35]. This research represents the first analysis of nationwide CRF08_BC data, infers a plausible genetic distance threshold 0.7% and established the transmission network. A previous study has been demonstrated that a lower genetic distance threshold can distinguish recent transmission in outbreak event and improve the probability that potential transmission partners share than epidemiological connection [20, 36]. Therefore, from a public health perspective, using this genetic distance threshold can not only obtain the maximum number of clusters in the CRF08_BC network but also better identify the recent partners in the growing transmission cluster.

Our study not only analysed the formation characteristics of networks but also explored mixing by risk category and it yielded significant new insights into HIV transmission among diverse populations. Not all cluster growth is equivalent, consequently, taking measures focused on the core group curbing risk behaviours are probable to reduce HIV infection transmission among other risk groups [7]. Moreover, our analysis used behavioural and epidemiologic information to investigate transmission patterns characteristics. Although CRF08_BC is mainly located in southwestern China, previous studies have described high frequencies CRF08_BC transmission in eastern China that may be attributed to high levels of immigrant labour in this region [35,37]. As a result, due to frequent population mobility, CRF08_BC has the possibility of a sharp increase as well in China.

HIV-1 CRF08_BC viral diversity varied across different risk populations in China [32,38]. Primarily, our research indicates that IDUs are primary transmission risk groups in the past. Notably, nowadays, the heterosexual contact has overtaken IDUs transmission routes as the greatest risk group for CRF08_BC infections nationwide (67.4 and 23.3%). It showed an obviously changes divergence of CRF08_BC transmission population which had resulted in the highest risk groups changed from IDUs to heterosexuals. Currently, the overall clustering rate of the network has decreased, but the results of the dynamics of network cluster growth trend indicated that not all cluster growth is equivalent [5,23]. Not surprisingly, by comparing the propagation networks of the two growth states, we found that the IDUs group in the network were slowly increasing or quiescent state in recent years, while the heterosexual transmission population is prominently active [1,10]. This suggests that our active/rapid growth network was the target that should be the focus of intervention in the future. Furthermore, we should strengthen the monitoring of heterosexual risk populations in the network, early diagnosis and treatment of infected people, and strengthen prevention and control management for uninfected people. For low-growth networks, it may also reflect the positive interventions for IDUs that have been taken in recent years, moreover, the effects of continuous advancement of treatment standards in China [20,39].

We found that clustering was associated with IDUs and heterosexual (male) group, young persons (aged 30–49 years), and lower education, suggesting that they are disproportionately involved in clusters associated with HIV transmission [16,20,40]. These factors also observed in the analysis of multiple potential transmission links. Previous studies report also has shown that nodes with more links in the network may play the role of “core population” who had a higher risk of transmission [41,42]. The level of behavioural intervention received by different educational schools is not balanced. Especially in China, influenced by traditional concepts, it is difficult to receive education about HIV/AIDS in the primary and middle schools. Therefore, some MSM/IDUs groups lack self-protection awareness due to their low education level. And in recent years, the MSM has gradually become a new group at higher risk for CRF08_BC transmission. This suggests that CRF08_BC has signs of transmission from the particular high-risk populations (IDUs and heterosexual) to the MSM population. Although MSM is less likely to be clustered at present, because the active transmission characteristics of the MSM population, it is easy to form “core population” with multiple potential transmission links. This part of the population has the ability to cause widespread transmission of CRF08_BC. Just like since CRF07_BC was first discovered, it has spread rapidly among China provinces through the MSM population [43]. Taken together, these results indicated that prioritizing IDUs, MSM groups, lower education and young person’s intervention was likely to be significantly effective in preventing the spread of CRF08_BC [5, 6, 44].

In this research, the IDUs infection among CRF08_BC likely originates from two predominant sources. Firstly, a large proportion of these groups were linked to other IDUs. Besides, we also found that many IDUs were linked to heterosexual women (45.4% of all links), which indicated that a substantial proportion of IDUs might be involved in transmission with heterosexual women. Moreover, our study found networking by heterosexual, with women much more likely to link to transmission networks with other IDUs. In contrast, there were fewer links with IDUs in the heterosexual men. Although we are not able to establish directionality of transmission from these sequences, suggesting that a portion of heterosexual women are related to IDUs transmission, these transmissions may represent that women are at risk for HIV infection through multiple routes. In the heterosexual transmitted infection population, we found that young (aged 30–49 years) and singlehood persons more commonly had multiple potential transmission partners. Notably, the vast majority of young IDUs (aged 30–39 years) were more likely to link to other heterosexual. This type of assortative mixing may be due to the reason that they are more likely to engage in Two-way propagation behaviour and they are disproportionately involved in clusters associated with HIV transmission [1,2,45]. Together, these findings suggested that prioritizing middle-aged persons of IDUs and heterosexual women infected may be the most effective strategies in preventing transmission of HIV. And identifying and controlling these “bridge population” is essential to curb the spread of HIV among the population. Previous research has also revealed that using molecular network features to target intervention can effectively reduce the spread of HIV [6].

Although the results of our research were robust, there are still some limitations. As sequence in this study may be affected by selection/sampling bias due to limited funding for HIV-1 sequence projects collected by molecular epidemiological survey and differences in data integrity by location and other characteristics. Nevertheless, to the best of our knowledge, this study has so far included the highest number of CRF08_BC strain sequences for transmission networks analysis in China. In the subsequent investigation, we expect this potential bias to decrease as we expand the number of participating regions and the integrity of the data. Additionally, the sample transmission category is based on self-reported information, we could not access available clinical following data, such as CD4 count, viral load, and accurate diagnosis time. As a result, there may have been other associations that we did not have power to detect. In the future, we plan to conduct more detailed analysis to understand these molecular epidemiological survey data.

The genetic transmission network surveillance and analysis for HIV could offer tools to understand transmission dynamics among various regions distribution and diverse risk population of CRF08_BC in China. As the scope and coverage of HIV transmission cluster surveillance continue to grow, we expect to be able to make effort inferences. These observations may also be significant for targeted prevention interventions, which could use the HIV genetic transmission networks, combined with clinical data and epidemiology data, to help us better identify potential transmission relationship and further make a better assessment of CRF08_BC transmission trends at the population level.

## Supplementary Material

Supplementary_Information.docx

## Data Availability

All data included in this study are available upon request by contact with the corresponding author.
