# Supplementary material for: Using molecular transmission networks to understand the epidemic characteristics of HIV-1 CRF08_BC across China
Source: Emerg Microbes Infect. 2021 Mar 22;10(1):497–506. doi: 10.1080/22221751.2021.1899056 (PMC7993390; doi:10.1080/22221751.2021.1899056)
Supplement: Supplementary_Information.docx [file TEMI_A_1899056_SM8615.docx]

**Using Molecular Transmission Networks to Understand the Epidemic Characteristics of HIV-1 CRF08_BC Across China**

**Running Title:** Genetic transmission networks reveal the transmission patterns of CRF08_BC

Kang Li^1,2^, Meiliang Liu^4^, Huanhuan Chen^3^, Jianjun Li^3^, Yanling Liang^2,4^, Yi Feng^2^, Hui Xing^2^, Yiming Shao^1,2,3#^

^1^Key Laboratory of Molecular Microbiology and Technology, Ministry of Education, College of Life Sciences, Nankai University, Tianjin, China.

^2^ State Key Laboratory for Infectious Disease Prevention and Control, National Center for AIDS/STD Control and Prevention, Chinese Center for Disease Control and Prevention, Beijing, China.

^3^Guangxi Center for Disease Prevention and Control, Nanning, Guangxi, China;

^4^ Guangxi Key Laboratory of AIDS Prevention and Treatment & Guangxi Universities Key Laboratory of Prevention and Control of Highly Prevalent Disease, School of Public Health, Guangxi Medical University, Nanning, Guangxi, China.

^#^Address correspondence to:

Yiming Shao

Division of Research on Virology and Immunology

National Center for AIDS/STD Control and Prevention

China CDC

Beijing 102206

China

E-mail: [yshao@bjmu.edu.cn](mailto:yshao@bjmu.edu.cn)

**Supplementary Information**


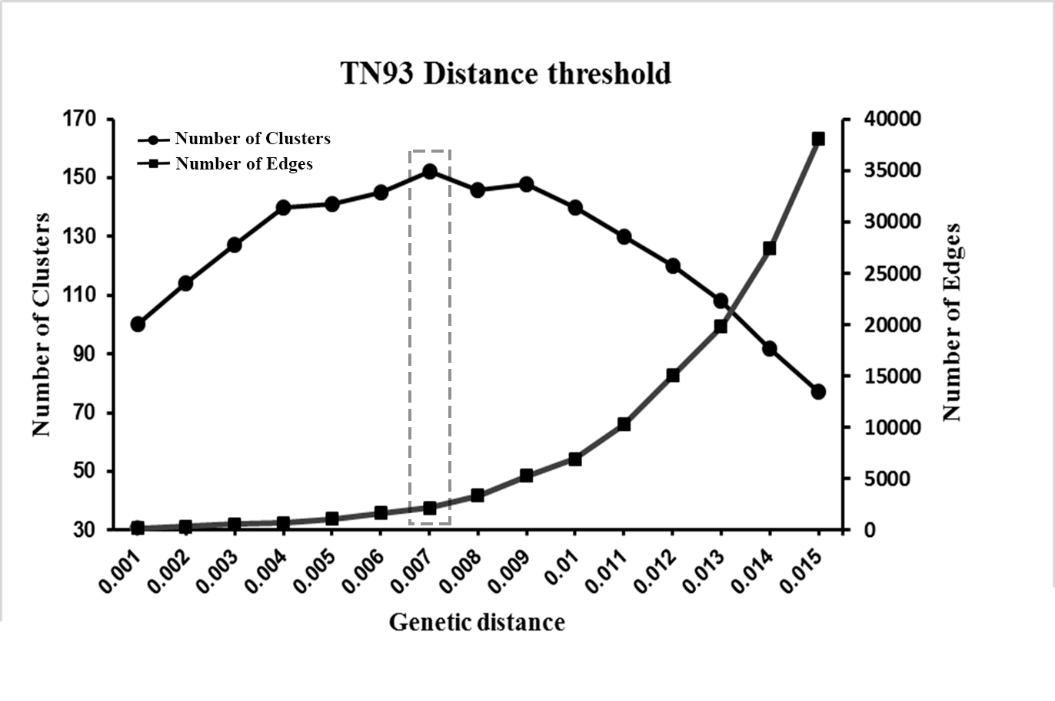


Figure S1. Determination of the optimal genetic distance threshold for HIV-1 CRF08_BC.

| **TableS1 Factors associated with clustering in the 08_BC transmission network in univariate logistic regression model** | | | | | | |
| --- | --- | --- | --- | --- | --- | --- |
| **Attribute** | **Category** | **Cases with a Genotype** | **Clustered in network, n (%)** | **Odds ratio** | **Odds ratio 95% CI** | ***P*-value** |
|  |  |  |  |  |  |  |
| All |  | 1,829 | 639(34.9) | — | | — |
| Transmission risk (birth sex) | Hetero (F) | 524 | 149(28.4) | — | — | — |
|  | Hetero (M) | 705 | 243(34.5) | 1.33 | 1.04-1.71 | 0.021 |
|  | Hetero (U) | 4 | 2(50.0) | 2.52 | 0.35-18.03 | 0.358 |
|  | MSM | 69 | 13(18.8) | 0.49 | 0.25-0.97 | 0.040 |
|  | IDUs | 426 | 207(48.6) | 2.38 | 1.82-3.11 | 0.000 |
|  | BT | 8 | 2(25.0) | 0.84 | 0.17-4.2 | 0.831 |
|  | Unknown | 93 | 23(24.7) | 0.83 | 0.5-1.37 | 0.463 |
| Region | Central | 122 | 26(21.3) | — | — | — |
|  | North | 32 | 11(34.4) | 1.99 | 0.85-4.67 | 0.115 |
|  | East | 293 | 102(34.8) | 1.93 | 1.18-3.17 | 0.009 |
|  | Southwest | 1,382 | 500(36.2) | 2.04 | 1.31-3.2 | 0.002 |
| Sampling Year | 2000-2007 | 304 | 193(63.5) | — | — | — |
|  | 2008-2011 | 483 | 173(35.8) | 0.32 | 0.24-0.43 | 0.000 |
|  | 2012-2015 | 695 | 147(21.2) | 0.15 | 0.11-0.21 | 0.000 |
|  | 2016-2017 | 347 | 126(36.3) | 0.33 | 0.24-0.45 | 0.000 |
| Education level | Primary school | 471 | 174(36.9) | — | — | — |
|  | Middle school | 685 | 234(34.2) | 0.69 | 0.68-0.87 | 0.002 |
|  | High school | 302 | 111(36.8) | 0.86 | 0.86-1.16 | 0.327 |
|  | College or higher | 190 | 55(28.9) | 0.59 | 0.61-0.87 | 0.006 |
|  | Unknown | 181 | 65(35.9) | 0.81 | 0.83-1.18 | 0.307 |
| Age at diagnosis | <20 | 61 | 16(26.2) | — | — | — |
|  | 20-29 | 505 | 148(29.3) | 1.17 | 0.64-2.13 | 0.617 |
|  | 30-39 | 459 | 190(41.4) | 1.99 | 1.09-3.62 | 0.025 |
|  | 40-49 | 316 | 129(40.8) | 1.94 | 1.05-3.58 | 0.034 |
|  | 50-59 | 164 | 51(31.1) | 1.27 | 0.66-2.45 | 0.478 |
|  | ≥60 | 183 | 62(33.9) | 1.44 | 0.75-2.75 | 0.269 |
|  | Unknown | 141 | 43(30.5) | 1.23 | 0.63-2.42 | 0.541 |
| Marital status | Single | 793 | 275(34.7) | — | — | — |
|  | Married | 739 | 265(35.9) | 1.05 | 0.85-1.3 | 0.629 |
|  | Divorced or widowed | 120 | 34(28.3) | 0.75 | 0.49-1.14 | 0.172 |
|  | Unknown | 177 | 65(36.7) | 1.09 | 0.78-1.53 | 0.606 |

Abbreviations: Hetero, heterosexual; MSM, men who have sex with men; IDUs, intravenous drug users; BT, blood transfusion; Unknown, data are not available; CI, confidence interval.

| **TableS2 Factors associated with potential transmission links in univariate logistic regression model** | | | | | | | |
| --- | --- | --- | --- | --- | --- | --- | --- |
| **Attribute** | **Category** | **Total** | **1-3 Links, n (%)** | **≥4Links, n (%)** | **Odds ratio** | **Odds ratio 95% CI** | ***P*-value** |
|  |  |  |  |  |  |  |  |
| All |  | 639 | 441(69.0) | 198(31.0) | — | — | — |
| Transmission risk (birth sex) | Hetero (F) | 149 | 119(79.9) | 30(20.1) | — | — | — |
|  | Hetero (M) | 243 | 196(80.7) | 47(19.3) | 0.97 | 0.58-1.61 | 0.896 |
|  | Hetero (U) | 2 | 1(50.0) | 1(50.0) | 3.97 | 0.24-65.27 | 0.335 |
|  | MSM | 13 | 7(53.8) | 6(46.2) | 3.31 | 0.95-11.57 | 0.061 |
|  | IDUs | 207 | 97(46.9) | 110(53.1) | 4.50 | 2.77-7.3 | 0.000 |
|  | BT | 2 | 2(100.0) | 0(0.0) | 0.00 | N/A | 0.999 |
|  | Unknown | 23 | 19(82.6) | 4(17.4) | 0.84 | 0.26-2.64 | 0.759 |
| Region | Central | 26 | 20(76.9) | 6(23.1) | — | — | — |
|  | North | 11 | 10(90.9) | 1(9.1) | 0.33 | 0.04-3.16 | 0.338 |
|  | East | 102 | 70(68.6) | 32(31.4) | 1.52 | 0.56-4.16 | 0.411 |
|  | Southwest | 500 | 341(68.2) | 159(31.8) | 1.55 | 0.61-3.95 | 0.353 |
| Sampling Year | 2000-2007 | 193 | 100(51.8) | 93(48.2) | — | — | — |
|  | 2008-2011 | 173 | 113(65.3) | 60(34.7) | 0.57 | 0.38-0.87 | 0.009 |
|  | 2012-2015 | 147 | 127(86.4) | 20(13.6) | 0.17 | 0.1-0.29 | 0.000 |
|  | 2016-2017 | 126 | 101(80.2) | 25(19.8) | 0.27 | 0.16-0.45 | 0.000 |
| Education level | Primary school | 174 | 113(64.9) | 61(35.1) | — | — | — |
|  | Middle school | 234 | 164(70.1) | 70(29.9) | 0.58 | 0.38-0.9 | 0.014 |
|  | High school | 111 | 74(66.7) | 37(33.3) | 0.71 | 0.44-1.12 | 0.140 |
|  | College or higher | 55 | 38(69.1) | 17(30.9) | 0.59 | 0.31-1.15 | 0.122 |
|  | Unknown | 65 | 52(80.0) | 13(20.0) | 0.35 | 0.17-0.71 | 0.004 |
| Age at diagnosis | <20 | 16 | 11(68.8) | 5(31.3) | — | — | — |
|  | 20-29 | 148 | 103(69.6) | 45(30.4) | 0.73 | 0.25-2.18 | 0.575 |
|  | 30-39 | 190 | 116(61.1) | 74(38.9) | 5.14 | 1.85-14.32 | 0.002 |
|  | 40-49 | 129 | 82(63.6) | 47(36.4) | 1.31 | 0.45-3.76 | 0.622 |
|  | 50-59 | 51 | 42(82.4) | 9(17.6) | 0.54 | 0.15-2 | 0.359 |
|  | ≥60 | 62 | 48(77.4) | 14(22.6) | 1.11 | 0.35-3.5 | 0.861 |
|  | Unknown | 43 | 39(90.7) | 4(9.3) | 0.29 | 0.06-1.35 | 0.116 |
| Marital status | Single | 275 | 187(68.0) | 88(32.0) | — | — | — |
|  | Married | 265 | 180(67.9) | 85(32.1) | 1.00 | 0.7-1.44 | 0.335 |
|  | Divorced or widowed | 34 | 24(70.6) | 10(29.4) | 0.89 | 0.41-1.93 | 0.061 |
|  | Unknown | 65 | 50(76.9) | 15(23.1) | 0.64 | 0.34-1.2 | 0.000 |

Abbreviations: Hetero, heterosexual; MSM, men who have sex with men; IDUs, intravenous drug users; BT, blood transfusion; Unknown, data are not available; CI, confidence interval.
